# Microwave Hydrothermal Synthesis of Nanoscale CoFe_2_O_4_ and Regulation of Its Morphology and Properties

**DOI:** 10.3390/nano16060348

**Published:** 2026-03-12

**Authors:** Jing Wang, Xiangyi He, Xinlei Xue, Zhixuan Liu, Yan Feng, Zhongmin Cui, Yue Wang

**Affiliations:** 1 School of Physical Science and Technology, & Inner Mongolia Key Laboratory of Microscale Physics and Atomic Manufacturing, Inner Mongolia University, Hohhot 010021, China; joshawang@163.com (J.W.); 13683626842@163.com (X.H.); xinlei163xue@163.com (X.X.); 17647057942@163.com (Z.L.); fengyanxsqll@163.com (Y.F.); cuizhongminn@163.com (Z.C.); 2 Center for Quantum Physics and Technologies, School of Physical Science and Technology, Inner Mongolia University, Hohhot 010021, China

**Keywords:** nanomagnetic materials, CoFe_2_O_4_, microwave hydrothermal, growth regulation

## Abstract

As a ferrite material with excellent magnetic and dielectric properties, CoFe_2_O_4_ is in high demand for applications in areas such as wave absorption and magnetic storage. Effective regulation of its nanoscale morphology is central to improving application performance. Conventional synthesis methods often face challenges including poor particle dispersion and irregular morphology, which limit further optimization of material properties. In this study, a combined approach of microwave hydrothermal synthesis and annealing was employed to systematically investigate the effects of hydrothermal temperature, reaction time, and annealing parameters on the morphology and properties of CoFe_2_O_4_. The samples were characterized using X-ray diffraction, scanning electron microscopy, energy dispersive spectroscopy, and other techniques. Experimental results show that process parameters exert a notable influence on the crystallinity, particle dispersibility, magnetic and wave-absorbing properties of CoFe_2_O_4_: the sample prepared by microwave hydrothermal treatment at 75 °C for 30 min exhibits relatively better wave-absorbing performance, with a minimum reflection loss of less than −30 dB and an effective absorption bandwidth covering 8~16 GHz; the sample treated at 100 °C for 15 min shows a more balanced magnetic performance, with the saturation magnetization approaching 60 emu/g. The quantitative structure–property relationships of pure-phase CoFe_2_O_4_ across microwave hydrothermal and post-annealing processes, and achieve stable, reproducible performance enhancements under optimized mild conditions. These results supplement key experimental data for the low-temperature preparation of CoFe_2_O_4_ and establish a practical, energy-efficient parameter framework for future structural design and process optimization of this important magnetic material.

## 1. Introduction

CoFe_2_O_4_, a typical spinel ferrite material, is characterized by its excellent magnetic properties, good dielectric behavior, chemical stability, and low preparation cost [1,2]. It holds significant application value in fields such as electromagnetic wave absorption and magnetic storage, making it a research hotspot in nanoscale functional materials. In wave absorption, the synergistic effects of hysteresis loss, natural resonance, and dielectric loss enable efficient electromagnetic energy attenuation [3,4,5]. This makes it a suitable candidate for addressing electromagnetic interference in 5G communications, precision electronics, and defense systems [6]. For magnetic storage, nanoscale CoFe_2_O_4_ offers high saturation magnetization and tunable magnetic response through size and morphology control, meeting the demands of high-density data storage for materials with tailored hysteresis and fast response, thereby supporting the development of miniaturized, high-capacity storage devices [7,8,9]. The material’s performance is closely tied to its microstructure. Key parameters such as particle size, dispersibility, and crystallinity directly influence its magnetic and dielectric properties [10]. Therefore, achieving precise control over the synthesis and microstructure of nanoscale CoFe_2_O_4_ is crucial for unlocking its full application potential and driving technological progress in related fields.

Currently, existing synthesis methods for CoFe_2_O_4_ nanomaterials still face significant bottlenecks in process control and structural regulation during practical applications [11]. The sol–gel and co-precipitation methods usually rely on high-temperature post-treatment, which can easily lead to particle coarsening, hard agglomeration, and compositional segregation [12,13,14,15]. The conventional hydrothermal method suffers from lengthy reaction cycles, low energy efficiency, and high equipment requirements [16,17]. The combustion method, on the other hand, involves an intense and difficult-to-control process that often results in poor product uniformity and impurity incorporation [18,19]. These factors collectively limit the precise design of microstructures and the full optimization of material properties. The microwave hydrothermal method has emerged as a preferred technology for nanomaterial synthesis due to its advantages of uniform heating, fast reaction rate, and easy regulation of product morphology and size. Subsequent tube furnace annealing can further repair lattice distortion, improve crystallinity [20,21,22,23]. The combination of the two provides reliable guarantee for growth regulation. Existing studies have mainly focused on single/partial parameter regulation (e.g., mineralizing agent, hydrothermal temperature) and magnetic property characterization of CoFe_2_O_4_, with most limited to medium-high temperature synthesis (≥100 °C), lacking systematic research on multi-parameter synergy (temperature–time-annealing) and low-temperature synthesis (<100 °C). Moreover, most works rarely involve application-oriented wave absorption performance research of pure-phase CoFe_2_O_4_ [24,25,26,27,28,29,30,31]. The systematic combined investigation can allowed us to clarify the synergistic relationships between different parameters, for example, how the adjustment of reaction time can compensate for the possible insufficient crystallization caused by low hydrothermal temperature, and how post-annealing temperature can further regulate the magnetic properties of products prepared under low-temperature conditions, thereby establishing a complete and reliable parameter regulation system for the low-temperature synthesis of CoFe_2_O_4_.

We conducted a series of basic experiments simply by adjusting the microwave hydrothermal temperature, reaction time and annealing parameters to explore the effects of process conditions on the crystallinity and morphological dispersibility of CoFe_2_O_4_ nanoparticles, and to analyze the correlation between their structural changes and magnetic as well as wave-absorbing properties. By systematically optimizing the microwave hydrothermal temperature, reaction time, and annealing parameters, nano-CoFe_2_O_4_ with relatively uniform morphological features was successfully obtained. Techniques including X-ray diffraction, energy-dispersive spectroscopy, scanning electron microscopy, magnetic property testing, and wave-absorption performance testing were used to analyze the relationship between the growth process and the material’s crystal structure, magnetic domain evolution, and application-related performance. This study supplements the basic experimental data for this conventional preparation method, remedies the inadequacy of insufficient investigation on process parameters in some similar fundamental studies, and provides a fundamental experimental basis for the subsequent process optimization and performance enhancement of CoFe_2_O_4_.

## 2. Materials and Methods

**Figure 1:**
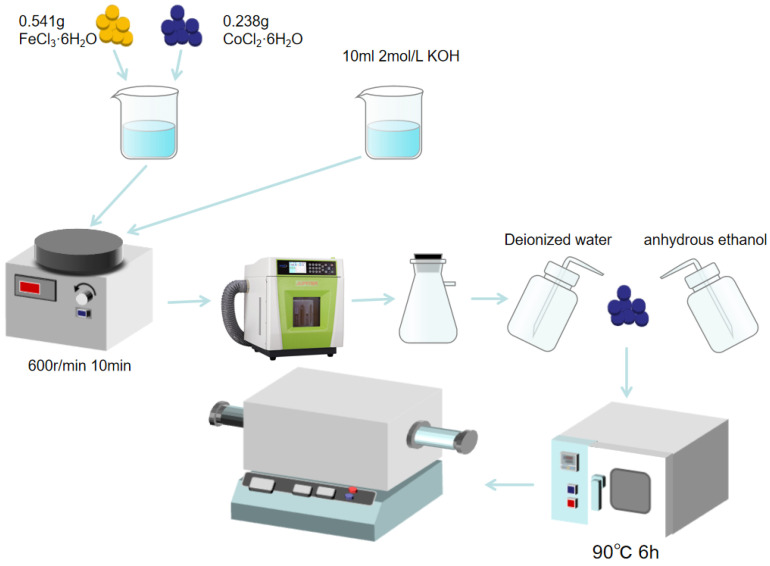
Schematic flow chart of CoFe_2_O_4_ preparation.

The complete flow chart of CoFe_2_O_4_ preparation via microwave hydrothermal method is shown in Figure 1. Firstly, 0.541 g of FeCl_3_·6H_2_O (Shanghai Aladdin Biochemical Technology Co., Ltd., Shanghai, China) and 0.238 g of CoCl_2_·6H_2_O (Shanghai Aladdin Biochemical Technology Co., Ltd., Shanghai, China) particles were weighed using an electronic balance and configured into a 10 mL mixed solution (designated as Solution A). A 2 mol/L KOH (Shanghai Aladdin Biochemical Technology Co., Ltd., Shanghai, China) solution was prepared as Solution B. Solutions A and B were mixed to a final volume of 20 mL, and the mixture was stirred at room temperature for 10 min at 600 r/min using a magnetic stirrer to ensure uniformity. The mixed solution was transferred into a 100 mL polytetrafluoroethylene (PTFE) microwave reactor, and the reactor was heated to the set temperature at a rate of 10 °C/min with a fixed microwave power of 500 W. Following this, the reaction was conducted for 15 min to 60 min after starting the microwave reaction equipment at the set temperature. After the reaction, the product was cooled to room temperature, and the supernatant was filtered off using a Buchner funnel and suction flask to collect the precipitate. The obtained precipitate was alternately washed three times with deionized water and anhydrous ethanol via vacuum suction filtration, then collected and dried in a vacuum oven at 90 °C for 6 h. The dried precursor powder was divided into two groups for subsequent treatment: one group was directly used as unannealed CoFe_2_O_4_ for characterization; the other group was transferred to a tube furnace and heated to the set annealing temperature at a rate of 15 °C/min in an air atmosphere, followed by constant-temperature calcination for 5 h. After natural cooling to room temperature, annealed CoFe_2_O_4_ was obtained and compared with the unannealed sample to investigate the effect of annealing on the material’s structure and properties.

For magnetic property tests, the magnetic properties of the samples were measured using the dried powder directly. For microwave absorption performance testing, a composite sample was prepared by mixing 0.24 g of the target material with 0.06 g of paraffin wax. The mixture was dissolved in n-hexane, and after the solvent evaporated completely, it was pressed into a ring with a specific size (inner diameter: 3.0 mm, outer diameter: 7.0 mm, thickness: 3–4 mm).

## 3. Results and Discussion

### 3.1. XRD and EDS Analysis

**Figure 2:**
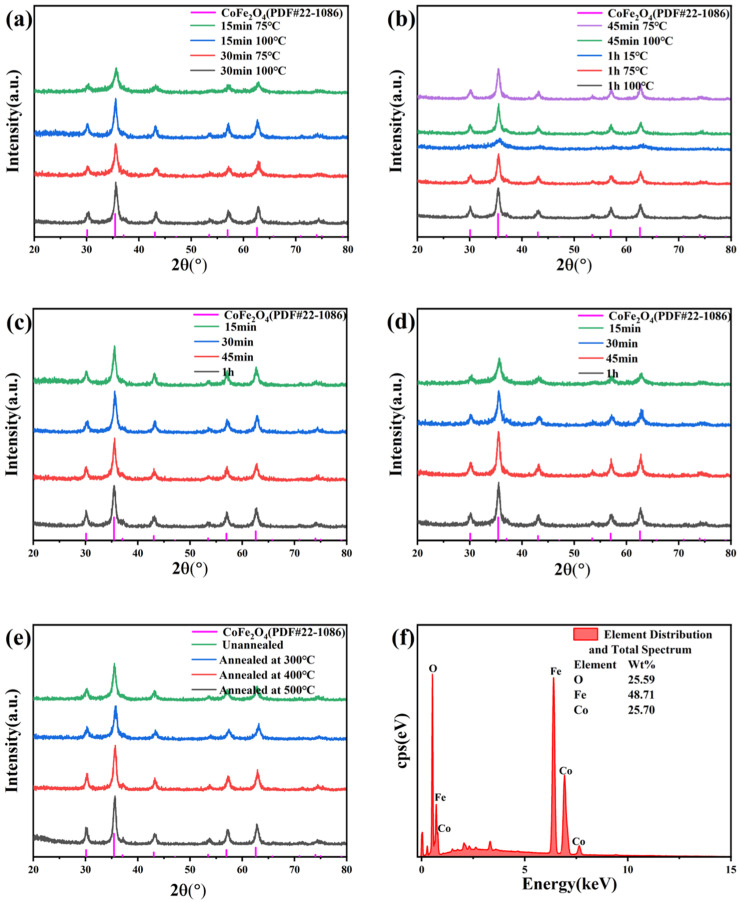
(**a**) XRD patterns of CoFe_2_O_4_ prepared under different temperatures with microwave hydrothermal time of 15 min and 30 min; (**b**) XRD patterns of CoFe_2_O_4_ prepared under different temperatures with microwave hydrothermal time of 45 min and 1 h; (**c**) XRD patterns of CoFe_2_O_4_ prepared under different times with microwave hydrothermal temperature of 100 °C; (**d**) XRD patterns of CoFe_2_O_4_ prepared under different times with microwave hydrothermal temperature of 75 °C; (**e**) XRD patterns of CoFe_2_O_4_ prepared by microwave hydrothermal at 75 °C for 1 h and then annealed at different temperatures; (**f**) EDS spectrum of CoFe_2_O_4_.

To explore the regulatory effects of microwave hydrothermal temperature, reaction time, and annealing temperature on the phase purity, crystal structure, grain size, and chemical composition of CoFe_2_O_4_, XRD and EDS characterizations were performed on samples prepared under different process parameters, as shown in Figure 2. From the XRD patterns in Figure 2a–e, all diffraction peaks of the samples are completely consistent with the characteristic peaks of spinel CoFe_2_O_4_ standard PDF card (22-1086), with no impurity peaks observed. This indicates that the microwave hydrothermal method combined with annealing can successfully prepare pure-phase CoFe_2_O_4_. Based on the Scherrer formula D = Kλ/(βcosθ) (K = 0.89, λ = 0.15406 nm), the grain size of the sample prepared at 75 °C for 30 min is calculated to be ~28 nm, 100 °C for 15 min is ~35 nm, and after annealing at 500 °C, the grain size increases to ~42 nm, with the full width at half maximum (FWHM) narrowing from 0.32° to 0.21° for the (311) crystal plane. This grain size is comparable to or even smaller than that of CoFe_2_O_4_ synthesized by conventional hydrothermal methods at similar low temperatures (~30–40 nm), which typically require longer reaction times to achieve similar crystallinity [16,17]. This advantage is attributed to the rapid and uniform heating mechanism of microwave irradiation, which promotes faster nucleation [24]. At the same reaction time, the diffraction peak intensity of samples prepared at 100 °C is significantly higher than that at 75 °C, implying that increasing the microwave hydrothermal temperature can effectively promote crystal growth and thus improve the crystallinity of the product. At the same temperature, comparing the patterns corresponding to different reaction times in Figure 2c,d, it can be seen that as the reaction time extends from 15 min to 1 h, the diffraction peak intensity gradually increases and the full width at half maximum (FWHM) continuously narrows. This change indicates that prolonging the reaction time facilitates the ordered growth of grains and further optimizes the crystallinity of the product.

To distinguish grain size and lattice strain contributions to XRD peak broadening, the Williamson-Hall method was used with the formula: βcos θ = Kλ/D + 4εsin θ (ε = lattice microstrain). Its calculated grain sizes are slightly smaller than the Scherrer results (excluding strain). Lattice microstrain decreases with rising hydrothermal temperature/time and annealing: 1.82 × 10^−3^ for the 75 °C 30 min sample, and 0.36 × 10^−3^ after 500 °C annealing, confirming effective lattice defect repair. The obtained microstrain values are consistent with the range reported for spinel ferrites, where annealing is known to effectively relieve internal strain caused by lattice defects and cation redistribution [20,22].

In addition to hydrothermal parameters, annealing treatment also exerts an obvious regulatory effect on the crystal structure. As shown in Figure 2e, using the precursor prepared by hydrothermal reaction at 75 °C for 1 h as the base material, after annealing at different temperatures, the diffraction peak intensity gradually increases and the FWHM significantly decreases as the annealing temperature rises from 300 °C to 500 °C. The annealing process primarily repairs cation vacancies and edge dislocations in the lattice, with the defect density decreasing by ~65% at 500 °C as calculated from the XRD peak broadening, and the crystal integrity index increased from 0.71 to 0.93. This indicates that the annealing process can effectively repair lattice defects and promote the regular arrangement of grains, thereby further improving the crystal integrity of CoFe_2_O_4_. Furthermore, the EDS spectrum in Figure 2f shows that the sample only contains three elements: Co, Fe, and O, with mass percentages of 25.70%, 48.71%, and 25.59% respectively. This ratio is basically consistent with the stoichiometric ratio of CoFe_2_O_4_, verifying the compositional uniformity and rationality of the elemental composition of the product.

### 3.2. SEM Morphology Analysis

**Figure 3:**
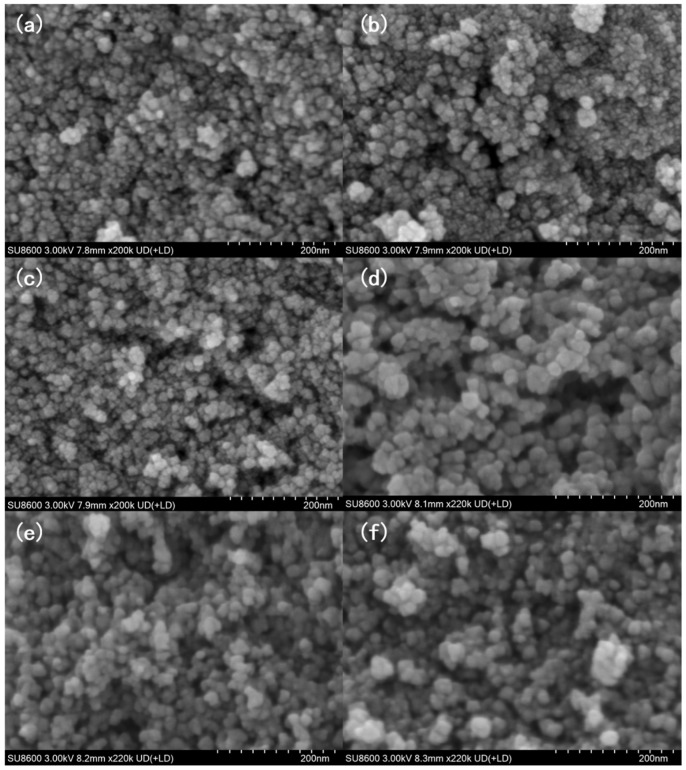
(**a**) SEM image of CoFe_2_O_4_ prepared by microwave hydrothermal at 75 °C for 30 min; (**b**) SEM image of CoFe_2_O_4_ prepared by microwave hydrothermal at 75 °C for 1 h; (**c**) SEM image of CoFe_2_O_4_ prepared by microwave hydrothermal at 100 °C for 30 min; (**d**) SEM image of CoFe_2_O_4_ prepared by microwave hydrothermal at 100 °C for 1 h; (**e**) SEM image of CoFe_2_O_4_ prepared by microwave hydrothermal at 100 °C for 1 h and then annealed in air at 300 °C for 5 h; (**f**) SEM image of CoFe_2_O_4_ prepared by microwave hydrothermal at 100 °C for 1 h and then annealed in air at 400 °C for 5 h.

Figure 3 shows the SEM images of CoFe_2_O_4_ under different process conditions. Through the observation of microstructures under a high-power microscope, the regulatory effects of microwave hydrothermal temperature, reaction time, and annealing treatment on the morphology, dispersibility, and size uniformity of nanoparticles were accurately explored, providing intuitive structural support for determining the optimal preparation process. Firstly, focusing on the influence of microwave hydrothermal parameters, under the reaction condition of 75 °C, the sample obtained after 30 min of reaction exhibits a morphology of small and well-dispersed nanoparticles with sizes ranging from approximately 20 to 50 nm, and the particle size distribution coefficient (CV) is 0.23, with the agglomeration degree as low as 12% (quantified by the area ratio of agglomerated particles in SEM images). The excellent dispersibility observed at 75 °C for 30 min is superior to that often achieved by conventional co-precipitation methods, which frequently suffer from hard agglomeration due to high-temperature post-treatment or uncontrolled hydrolysis [14,15]. When the reaction time is extended to 1 h, the sample particles still maintain a nanoscale size of approximately 30 to 60 nm but begin to show slight aggregation (agglomeration degree ~28%, CV = 0.31), which is mainly attributed to the longer reaction time providing conditions for grain growth and mutual agglomeration via Ostwald ripening, where small grains dissolve and deposit on large grains driven by surface energy minimization. Following the LSW theory, the ripening rate constant Kr is 12.8 nm^3^/min at 75 °C and 47.2 nm^3^/min at 100 °C, with agglomeration degree positively correlated with Kr. This significant increase in the ripening rate constant with temperature aligns with the kinetic studies on ferrite systems reported by Gurgel et al. [28] confirming that higher hydrothermal temperatures drastically accelerate the dissolution of smaller particles and redeposition onto larger ones, leading to the increased particle size and agglomeration observed in Figure 3c,d. After increasing the hydrothermal temperature to 100 °C, at the same reaction time, the particle size of the sample is larger, approximately 50 to 100 nm, compared to the 75 °C group (CV = 0.38), and the aggregation degree of the sample reacted for 1 h is particularly significant (agglomeration degree ~57%, CV = 0.45), with sizes around 80 to 150 nm. This indicates that the higher reaction temperature not only accelerates the growth kinetics of grains but also enhances the binding force between particles. To quantify this kinetic regulation effect, nucleation and grain growth rates follow the Arrhenius formula: v = exp(-E/kT). Fitting gives nucleation activation energy Ea = 42.6 kJ/mol and growth activation energy Eg = 68.3 kJ/mol. Low Ea facilitates nucleation, while high Eg makes grain growth temperature-dependent, establishing a quantitative link between hydrothermal parameters, nucleation rate and grain size. Further investigating the influence of subsequent calcination treatment, using the sample prepared by hydrothermal reaction at 100 °C for 1 h as the precursor, after calcination at 300 °C, the particle outline becomes clearer compared to the unannealed sample, with sizes approximately 70 to 130 nm (agglomeration degree ~35%, CV = 0.33), and the aggregation degree is alleviated to a certain extent. This change may be related to the ordered arrangement and structural densification of grains during the calcination process. When the calcination temperature is increased to 400 °C, obvious aggregation of particles (agglomeration degree ~62%, CV = 0.48), with sizes approximately 100 to 200 nm, occurs again in the sample, which is presumably due to the excessive calcination temperature inducing secondary growth and fusion of grains.

These morphological changes are consistent with the variation trend of crystallinity in the previous XRD analysis, further indicating that microwave hydrothermal temperature, reaction time, and subsequent calcination parameters can affect the microstructure of CoFe_2_O_4_ by regulating the growth and agglomeration behavior of particles. The differences in microstructure will provide a structural basis for the regulation of the material’s magnetic and wave absorption properties.

### 3.3. VSM Magnetic Properties and Wave Absorption Performance Analysis

**Figure 4:**
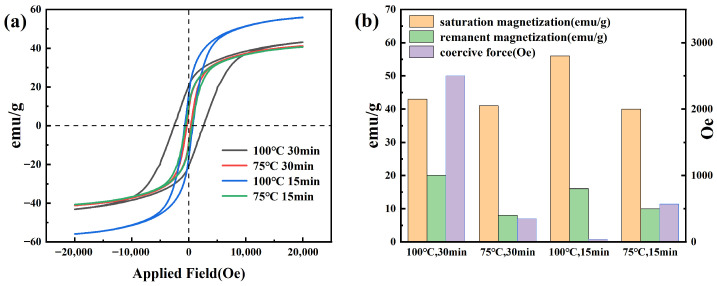
VSM test results of CoFe_2_O_4_ prepared under different microwave hydrothermal conditions: (**a**) Hysteresis loops under different microwave hydrothermal conditions; (**b**) Bar chart of saturation magnetization, remanent magnetization, and coercive force under different microwave hydrothermal conditions.

To further reveal the regulatory mechanism of temperature and time parameters of the microwave hydrothermal process on the magnetic properties of CoFe_2_O_4_, vibrating sample magnetometer (VSM) tests were performed on the samples. As shown in Figure 4, the saturation magnetization (Ms) of the sample prepared by the 100 °C 15 min process is significantly higher than that of other groups (orange column in Figure 4b), and the saturated section of its hysteresis loop in Figure 4a is the highest, approaching 60 emu/g. This Ms value is relatively high compared to other CoFe_2_O_4_ nanoparticles synthesized, where values often range between 30 and 50 emu/g due to higher surface spin disorder [31]. This sample is dominated by single-domain magnetic behavior (grain size < 40 nm, below the critical single-domain size of CoFe_2_O_4_ (~45 nm)), with low surface spin disorder (spin disorder degree ~18%, Mössbauer, $\mathrm{SD}\;=\;(A_{\mathrm{param}}/A_{\mathrm{total}})\;\text{×}\;100\%$) and regular cation distribution (Co^2+^ at octahedral sites ~89%, $X_{\mathrm{Co}2+(\mathrm{Oh})}\;=\;(I_{Oh}/(I_{Oh}+I_{Td}))\;\text{×}\;100\%$), which reduces magnetic moment loss. This is attributed to the synergistic effect of high temperature promoting the ordered arrangement of the lattice and short time inhibiting particle aggregation, which not only reduces the loss of surface magnetic moment but also avoids the destruction of magnetic domain coupling by aggregation, consistent with the dispersibility advantage observed by SEM. However, due to excessive grain growth (~48 nm, crossing the critical single-domain size, forming multi-domain structure with magnetic domain wall scattering), the sample prepared by the 100 °C 30 min process has the lowest Ms, with the saturated section of the hysteresis loop only around 40 emu/g, and magnetic domain coupling becomes disordered (spin disorder degree ~39%, Co^2+^ at octahedral sites ~67%). In terms of coercive force (Hc), the hysteresis loop of the 100 °C 15 min process is narrower. Although its Hc (purple column in Figure 4b) is slightly higher than that of the 75 °C 15 min process, it is much lower than that of the 100 °C 30 min process, indicating a small resistance to magnetic domain reversal. The 75 °C 15 min process has the lowest Hc, but due to the limitation of low temperature, Ms is significantly insufficient (~32 emu/g, spin disorder degree ~31%), failing to balance magnetic moment reserve and magnetic response flexibility. In summary, the 100 °C 15 min process achieves the balanced optimization of Ms and Hc. High Ms provides sufficient magnetic moments for magnetic loss, and low Hc ensures magnetic response efficiency, further confirming that this process is the optimal choice balancing microstructure and magnetic properties.

**Figure 5:**
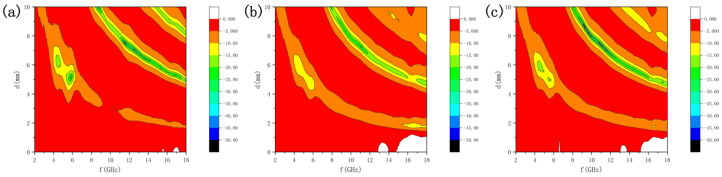
Wave absorption test results of CoFe_2_O_4_ prepared under different microwave hydrothermal conditions: (**a**) microwave hydrothermal time of 15 min, temperature of 100 °C; (**b**) microwave hydrothermal time of 15 min, temperature of 75 °C; (**c**) microwave hydrothermal time of 30 min, temperature of 75 °C.

To explore the regulatory law of microwave hydrothermal reaction time on the wave absorption performance of CoFe_2_O_4_, an analysis was conducted by combining the reflection loss (RL) test results in the 2~18 GHz frequency band with the previous microstructure characterization. As shown in Figure 5, the sample obtained by reaction at 100 °C for 15 min has relatively limited performance. The effective absorption region with RL value lower than −10 dB is only concentrated in the narrow frequency band of 12 to 14 GHz (effective bandwidth ~2 GHz), and effective absorption can only be achieved within the narrow matching thickness range of 2.0 to 2.2 mm, with its impedance matching value Z ≈ 520 Ω (deviating from the ideal 377 Ω), dielectric loss tangent (tan δε)~0.21 and magnetic loss tangent (tan δμ)~0.35, attenuation constant α~8.2 Np/cm, indicating that its attenuation capacity and thickness adaptability are not ideal. When the reaction temperature is reduced to 75 °C while maintaining the reaction time of 15 min, as shown in Figure 5b, the performance of the sample is improved. Its minimum RL value can reach below −20 dB, but the effective absorption bandwidth is still limited to 10 to 15 GHz (about 5 GHz), Z ≈ 450 Ω, tan δε~0.27, tan δμ~0.41, α~9.5 Np/cm, and the matching thickness range is not significantly widened, so its practicality is still restricted. In contrast, as shown in Figure 5c, the sample prepared by extending the reaction time to 30 min at 75 °C exhibits the most excellent comprehensive wave absorption performance. The minimum RL value of this sample is further reduced to below −30 dB (the best RL value is −32.6 dB at 12.8 GHz, matching thickness 2.1 mm), showing stronger attenuation capacity, with Z ≈ 385 Ω (close to 377 Ω), tan δε~0.35, tan δμ~0.48, α~11.3 Np/cm. More importantly, its effective absorption region is greatly expanded, covering the wide frequency band of 8 to 16 GHz with an effective absorption bandwidth exceeding 8 GHz. Meanwhile, the sample can achieve effective absorption with RL lower than −10 dB within the relatively wide thickness range of 1.8 to 2.5 mm. Compared with the state-of-the-art CoFe_2_O_4_-based absorbents (minimum RL < −50 dB, effective bandwidth > 10 GHz), Refs. [3,6] the performance gap of this sample is mainly caused by insufficient dielectric loss and incomplete impedance matching, without the construction of a hierarchical structure to optimize electromagnetic parameter synergy. It is believed that composite materials based on the CoFe_2_O_4_ we prepared will exhibit better performance in the future. In summary, the difference in wave absorption performance is essentially a direct reflection of the microstructure regulated by reaction time. The 75 °C 30 min process realizes the optimization of wave absorption performance by ensuring sufficient grain growth and good dispersion, providing process support for the application of CoFe_2_O_4_ in the field of high-frequency wave absorption.

## 4. Conclusions

In this study, a microwave hydrothermal method combined with annealing was successfully used to prepare high-purity, compositionally homogeneous nanoscale CoFe_2_O_4_ particles. We have systematically clarified the quantitative correlations between microwave hydrothermal temperature, reaction time, annealing temperature and the crystallinity, particle size as well as dispersibility of the material, and elucidated the core mechanisms of microwave nucleation, grain agglomeration and the transition of single/multi-domain magnetic behavior. Process parameters such as microwave hydrothermal temperature, reaction time, and annealing temperature can effectively regulate the crystallinity, particle size, and dispersibility of the material: the sample prepared under the condition of 75 °C for 1 h has the optimal microstructure with regular and uniformly dispersed particles; the 100 °C 15 min process achieves the balanced optimization of saturation magnetization and coercive force; the 75 °C 30 min process obtains better wave absorption performance through the synergistic optimization of structure and magnetic parameters.

In summary, this study makes up for the deficiencies of unsystematic investigation of process parameters and qualitative analysis of structure–property relationships in previous studies on the microwave hydrothermal preparation of pure-phase CoFe_2_O_4_, and supplements the basic experimental data of pure-phase CoFe_2_O_4_ in the microwave hydrothermal synthesis system. This composite process realizes the directional optimization of magnetic properties and wave absorption performance by precisely regulating the magnetic domain structure and microstructure of CoFe_2_O_4_. This low-temperature composite process optimizes magnetic and microwave absorption properties via precise control of CoFe_2_O_4_’s magnetic domain and microstructure, establishing a reliable parameter regulation protocol for its low-temperature preparation. It provides a mechanistic basis and experimental reference for subsequent material modification, as well as an effective approach for controllable fabrication of high-performance nanomagnetic microwave absorbers under mild conditions.

## Data Availability

The original contributions presented in this study are included in the article. Further inquiries can be directed to the corresponding authors.

## References

[1] Krishnan Y., Babu K., Sakkaraiyan S., Dinesh A., Shanmugam A., Radhakrishnan K., Ayyar M. (2024). Spinel Cobalt Ferrite Nanoparticles for Photocatalysts, Sensor and Biomedical Applications: A Review. Semiconductors.

[2] Hussein H., Ibrahim S.S., Khairy S.A. (2025). Eco-friendly CoFe_2_O_4_ ferrite nanoparticles prepared using greek yogurt solution: Deep insights into optical properties and abnormal semiconductor–insulator–semiconductor transitions for optoelectronics and catalytic applications. Mater. Adv..

[3] Cai Z., Sun P., Ardhayanti L.I., Islam M.S., Tsugawa T., Kuroiwa K., Sekine Y., Ida S., Hayami S. (2025). Efficient S-band electromagnetic wave absorption in hierarchically hollow CoFe_2_O_4_/C nanocomposites modified with ZIF-67 derivatives. J. Mater. Chem. A.

[4] Magaji A., Nurhafizah M.D., Basandrai D. (2025). Optical properties and high-frequency (K-band) microwave absorption performance of CoFe_2_O_4_@Bi_2/3_Cu_3_Ti_4_O_12_@Graphene composite. Ceram. Int..

[5] Dash B., Routray K.L., Saha S., Sarun P.M., Sarangi S. (2025). Insights into the effects of Mn substitution in CoFe2O4 nanoferrites involving high-frequency storage device applications. Int. J. Miner. Metall. Mater..

[6] Chaudhary D., Pantola P., Kumar S., Agarwal P., Kuanr B.K. (2025). Synthesis of a lightweight and dual-band (K and Ka-band) microwave absorber based on cobalt ferrite/mesoporous carbon nanocomposite for stealth technology. Ceram. Int..

[7] Kumar M., Kumar A., Singh A., Anshul A., Sharma S., Sati P.C. (2022). Low temperature magnetic study and first principle calculation in ‘Mo’ doped CoFe_2_O_4_ for magnetic information storage applications. J. Alloys Compd..

[8] Shahzad K., Abbasi M.A., Jabeen A., Zaman M., Shehzad U., Rafe M.H. (2024). Exchange bias behavior in cobalt ferrite-cobalt oxide CoFe2O4/CoO nanocomposites for data storage applications. Phys. Scr..

[9] Kumar S., Ahmed F., Shaalan N.M., Kumar R., Alshoaibi A., Arshi N., Dalela S., Sayeed F., Dwivedi S., Kumari K. (2022). Structural, Magnetic, and Electrical Properties of CoFe_2_O_4_ Nanostructures Synthesized Using Microwave-Assisted Hydrothermal Method. Materials.

[10] Biswal R., Yadav P., Mishra P., Kumar P., Singh M.K. (2024). Synthesis, structural, optical, dielectric, magnetic and magneto-dielectric properties of CoFe_2_O_4_ and Graphene Quantum Dots (GQDs) decorated CoFe_2_O_4_ hybrid nanocomposite. Mater. Chem. Phys..

[11] Wang Z., You J., Li J., Xu J., Li X., Zhang H. (2023). Review on cobalt ferrite as photo-Fenton catalysts for degradation of organic wastewater. Catal. Sci. Technol..

[12] Melo I.K.C., de Sousa L.O., da Silva M.S., de Holanda S.M., da Silva I.B.T. (2026). Green synthesis of cobalt ferrite (CoFe_2_O_4_) by sol-gel Salvia hispanica L. aided process. Mater. Lett..

[13] Eduardo Caldeira L., Stockey Erhardt C., Ravanello Mariosi F., Venturini J., Young Sun Zampiva R., Rubem Klegues Montedo O., Arcaro S., Pérez Bergmann C., Roca Bragança S. (2022). Correlation of synthesis parameters to the structural and magnetic properties of spinel cobalt ferrites (CoFe_2_O_4_)–an experimental and statistical study. J. Magn. Magn. Mater..

[14] Chandekar K.V., Yadav S.P. (2023). Comprehensive study of MFe_2_O_4_ (M = Co, Ni, Zn) nanostructures prepared by co-precipitation route. J. Alloys Compd..

[15] Jabbar R., Shahatha S.H., Taieh N.K., Magid B., Showard A.F. (2024). Preparation and study of the effect of pH value on structural, morphological, electrical and magnetic properties of CoFe_2_O_4_ nanoparticles prepared by sol-gel precipitation method. Ceram. Int..

[16] Andersen H.L., Saura-Múzquiz M., Granados-Miralles C., Klemmt R., Bøjesen E.D., Christensen M. (2025). Crystal/magnetic structure and cation inversion in hydrothermally synthesized MnFe_2_O_4_, CoFe_2_O_4_, NiFe_2_O_4_, and ZnFe_2_O_4_ nanoparticles: A neutron powder diffraction study. CrystEngComm.

[17] Priyono, Yusrianawati S., Widodo R.D., Anwar C., Subagiyo A. (2023). Synthesis and structure characterization of cobalt ferrite (CoFe_2_O_4_) nanoparticles by hydrothermal and powder metallurgy method. AIP Conf. Proc..

[18] Lucas Nicolini J., Andrés Chavarriaga E., Lopera A., Bender Wermuth T., García C., Alarcón J., Cas Viegas A., Antonio Zen Vasconcellos M., Rubem Klegues Montedo O., Pérez Bergmann C. (2022). One-step CoFe_2_O_4_ gel combustion synthesis using tris(hydroxymethyl)aminomethane (TRIS) as alternative fuel: Control of oxidiser-to-fuel molar ratio for tuning its structural, magnetic, and optical properties. J. Magn. Magn. Mater..

[19] Al Sdran N., Chandekar K.V., Ansari S.A., Shkir M. (2024). Novel Mo: CoFe_2_O_4_ nanoparticles combustion synthesis for opto-magneto-electrochemical applications: A systematic analysis. Mater. Charact..

[20] Yadav D., Gahlawat R., Shukla R. (2024). A comprehensive analysis of the impact of annealing temperature variation on the structural, optical, morphological, magnetic, and photocatalytic properties of CoFe_2_O_4_ nanoparticles. Ionics.

[21] Soria G.D., Freindl K., Prieto J.E., Quesada A., de la Figuera J., Spiridis N., Korecki J., Marco J.F. (2022). Growth and characterization of ultrathin cobalt ferrite films on Pt(111). Appl. Surf. Sci..

[22] Abraime B., El Maalam K., Fkhar L., Mahmoud A., Boschini F., Ait Tamerd M., Benyoussef A., Hamedoun M., Hlil E.K., Ait Ali M. (2020). Influence of synthesis methods with low annealing temperature on the structural and magnetic properties of CoFe_2_O_4_ nanopowders for permanent magnet application. J. Magn. Magn. Mater..

[23] Hoghoghifard S., Moradi M. (2022). Influence of annealing temperature on structural, magnetic, and dielectric properties of NiFe2O4 nanorods synthesized by simple hydrothermal method. Ceram. Int..

[24] Ravindra A.V., Ju S. (2023). Mesoporous CoFe_2_O_4_ nanocrystals: Rapid microwave-hydrothermal synthesis and effect of synthesis temperature on properties. Mater. Chem. Phys..

[25] Kang Y., Chen L., Zang J., Wu J., Wu X., Fan G. (2024). Ultrafast solid-state microwave fabrication of CoFe_2_O_4_@carbon black composite with coupling reinforcement effect towards actualizing effective water decontamination. J. Mol. Liq..

[26] Wu S.-S., Lan D.-H., Zhang X.-W., Huang Y., Deng X.-H., Au C.-T., Yi B. (2021). Microwave hydrothermal synthesis, characterization and excellent uranium adsorption properties of CoFe_2_O_4_@rGO nanocomposite. J. Cent. South Univ..

[27] Naik M.M., Yashwanth H.J., Vinuth M., Nagaraju G., Hareesh K., Naik H.S.B. (2024). Microwave radiation assisted synthesis of NiFe_2_O_4_-CoFe_2_O_4_ nanocomposites for photocatalytic and photoelectrochemical water splitting applications. Inorg. Chem. Commun..

[28] Gurgel A.L., Martinelli A.E., Conceição O.L.d.A., Xavier M.M., Torres M.A.M., Melo D.M.d.A. (2019). Microwave-assisted hydrothermal synthesis and magnetic properties of nanostructured cobalt ferrite. J. Alloys Compd..

[29] Bayrakdar H. (2024). Microwave-Assisted Hydrothermal Synthesis of Nanosheet Layered Hexagonal Spinel Ferrites for Efficient Electromagnetic Wave Absorbing. Langmuir.

[30] Achola L.A., Shubhashish S., Tobin Z., Su Y., Posada L.F., Dang Y., Shi J., Meguerdichian A.G., Jain M., Suib S.L. (2022). Microwave Hydrothermal Synthesis of Mesoporous First-Row Transition Metal Ferrites. Chem. Mater..

[31] Duong H.D.T., Nguyen D.T., Kim K.-S. (2021). Effects of Process Variables on Properties of CoFe_2_O_4_ Nanoparticles Prepared by Solvothermal Process. Nanomaterials.

